# Nephrocalcinosis fortuitously discovered: the role of surreptitious self administration of diuretics

**DOI:** 10.22088/cjim.15.1.22

**Published:** 2024

**Authors:** Nery Sablón-González, Liliana Morán-Calcedo, Maria Belen Alonso-Ortiz, Yanet Parodis-López, Angelica Laurin, Emmanuel Andrès, Noel Lorenzo-Villalba

**Affiliations:** 1 Department of Nephrology, Dr Negrin University Hospital, Gran Canaria, Spain; 2 Department of Nephrology, University Hospital del Tajo, Madrid, Spain; 3 Department of Internal Medicine, Dr Negrin University Hospital, Gran Canaria, Spain; 4 Avericum Hemodyalisis Center, Chiclana de la Frontera, Spain; 5 Barriom Atlantico Primary Care Center, Las Palmas, Spain; 6 Department of Internal Medicine, Strasbourg University Hospital, Strasbourg, France

**Keywords:** Nephrocalcinosis, Hypokalemia, Furosemide

## Abstract

**Background:**

Furosemide is a drug widely used for several medical conditions and could be used without medical prescription. Furosemide-related nephrocalcinosis can occur regardless of age, although the risk is higher in premature infants. The defining characteristic of nephrocalcinosis is generalized calcium deposition in the kidney. The most useful imaging studies for evaluation are ultrasonography and computed tomography (more effective in detecting calcification).

**Case Presentation:**

A 32-year-old woman with a history of depressive syndrome was admitted for evaluation of fortuitously discovered nephrocalcinosis and hypokalemia. The studies performed revealed the presence of a metabolic alkalosis with discrete hyperreninism/hyperaldosteronism but normal ratio, normotension and urinary study showed elevated sodium, chloride, potassium and calcium fluctuating in different determinations. Surreptitious diuretic intake was suspected and urine analysis revealed doses equivalent to 80-120 mg. The patient was advised to discontinue all diuretic treatment; she was adequately supplemented with potassium and she was followed-up in outpatient clinics. During the follow-up, clinical and analytical improvement was noted, which led to the discontinuation of supplementation.

**Conclusion:**

Surreptitious diuretic intake is a clinical condition to rule out in patients with chronic hypokalemia, metabolic alkalosis with elevated urinary sodium and chloride. The relation between surreptitious diuretic intake and nephrocalcinosis has not been fully elucidated in adults.

Furosemide is a drug widely used for several medical conditions and could be used without medical prescription for weight control or edema in some countries. Thus, furosemide could be inappropriate used for a long period before misused and its consequences are discovered. Nephrocalcinosis associated with furosemide therapy have been described in premature infants (1); however, it is still not clear whether nephrocalcinosis can occur under this circumstance in adults. One study in rats showed that nephrocalcinosis occurred in all age groups following 2 weeks of furosemide treatment (2), this might suggest that furosemide-related nephrocalcinosis can occur regardless of age, although the risk is higher in premature infants. 

## Case Presentation

A 32-year-old female patient was referred for evaluation of a fortuitously discovered nephrocalcinosis and hypokalemia. Her medical history was relevant for an anxious depressive syndrome after her first delivery 6 years ago. She smokes 30 cigarettes/day, no alcohol consumption and no drug use. She was allergic to penicillin. 

**Figure 1:**
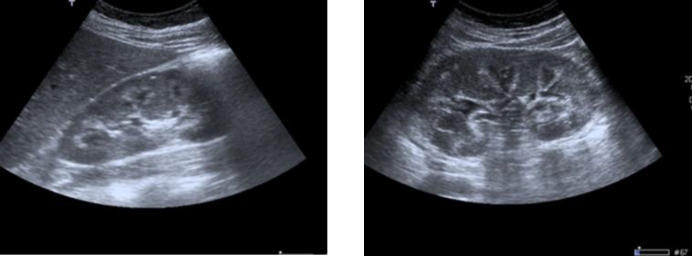
Renal echography. Right kidney (right) and left kidney (left)

**Figure 2:**
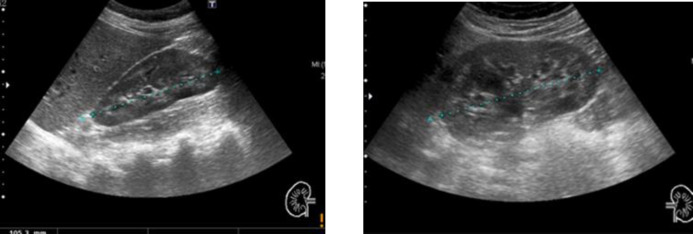
Previous renal echography. Right kidney (right) and left kidney (left)

The patient reported diarrhea over the past 4 or 5 years, 3 to 5 times a day, mainly diurnal, frequent nausea and vomiting, abundant fluid intake, occasional paresthesia and weight loss of approximately 5 kg. The ultrasound performed by her primary care physician described both kidneys of preserved size and morphology with adequate thickness and medullary cortical differentiation, highlighting the presence of a hyperechogenic rim surrounding all medullary pyramids bilaterally compatible with medullary nephrocalcinosis (figure 1). However, the patient had undergone a renal ultrasound 6 years earlier which had been reported as normal (Figure 2).

On physical examination, her height was 166 cm, weight 61 kg, blood pressure 110/70 mmHg and heart rate was 81 beats/min, she was afebrile, and oxygen saturation was 98% on room air. Mucous membranes were moist and well-colored. No skin lesions were found. Heart sounds were regular without murmurs or rubs, pulses were normal, and there was no edema. Lungs were clear. The abdomen was tender but not distended, and without palpable masses

In the presence of this fortuitously discovered nephrocalcinosis, a complete blood test was ordered. Thyroid, parathyroid function and hemoglobinA1c were within the normal range. Serology for HIV, HBV, HCV and syphilis were negative. Serum protein electrophoresis and serum immunoglobulins were normal. Quantiferon and Mantoux negative. Alpha-antitrypsin, cupper and ceruloplasmine were normal. Antinuclear antibodies, anti-DNA, soluble antinuclear antibodies, rheumatoid factor and angiotensin converting enzyme were negative. Renal function was normal as well as serum magnesium, total and corrected calcium, uric acid and vitamin D, however potassium was 2.8 mmol/L and chloride was 90 mmol/L.

**Figure 3:**
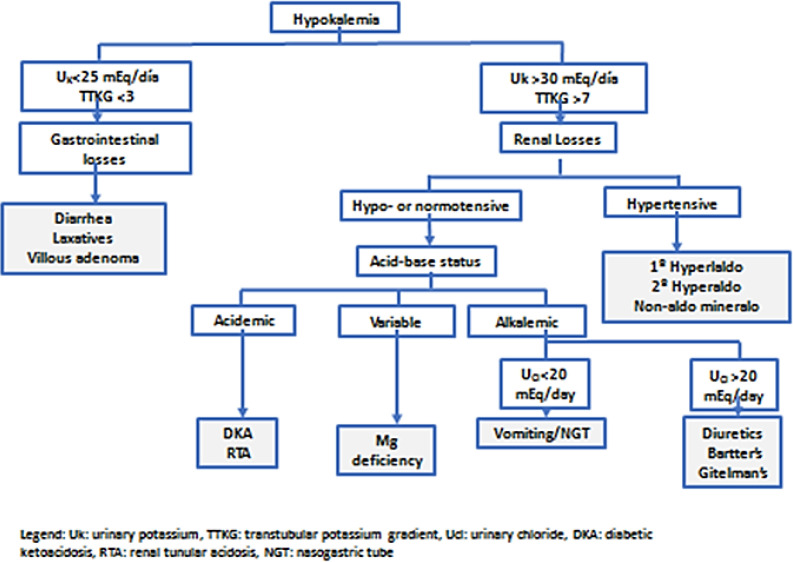
Approach to hypokalemia

In this context of nephrocalcinosis with hypokalemia and normotension, venous blood gases were ordered showing metabolic alkalosis (pH 7.47; pCO2 46.4 mmHg; HCO3 32.6 mmol/L) found. In this analytical context, we determined the transtubular potassium gradient (TTKG) (figure 3). The determination of potassium in isolated urine samples can be influenced by the concentration or dilution state of the urine. In our patient the TTKG=K (urine) x Osm (plasma) / K (plasma) x Osm (urine) showed values > 7 (17.22 x 279 / 2.1 x 247 = 9.26). The patient showed slightly elevated renin and aldosterone levels but with normal ratio, normotension and abdominal scanner that did not describe lesions at the level of the adrenal glands. 

No protein, red blood cells or crystals were found in urinalysis. A 24-hour urine test showed normal magnesium and oxalates but elevated levels of potassium, sodium, chloride and calcium. In this context, the diagnostic challenge was to distinguish between tubulapathy (Bartter's syndrome) and chronic surreptitious diuretic intake. 

Renal production of prostaglandin E2 was normal. A urinary determination of diuretics was performed describing the presence of furosemide at doses equivalent to 80-120 mg in a patient who was not under such medication. No other diuretics were found in the analyzed sample (thiazides, spironolactone). In this context, the patient was re-interviewed and she confirmed that she had been taking furosemide regularly for 5 years in doses ranging from 120-160 mg/d, which was difficult to corroborate upon questioning. 

The objective of this self-medication was weight loss in a patient who manifested body morphology disorders. We assumed that the clinical picture was related to the surreptitious intake of diuretics. The patient was advised to discontinue all diuretic treatment; she was adequately supplemented with potassium and was followed-up in outpatient clinics. During the follow-up, clinical and analytical improvement was noted, which led to the discontinuation of supplementation. This clinical evolution would support the initial diagnostic hypothesis.

## Discussion

Several clinical conditions may provoke nephrocalcinosis: Alport syndrome, Bartter syndrome, chronic glomerulonephritis, familial hypomagnesemia, medullary cancellous kidney, primary hyperoxalurias, kidney transplant rejection, renal tubular acidosis (RTA), and renal cortical necrosis. Other possible causes of nephrocalcinosis include: ethylene glycol toxicity, hypercalcemia due to hyperparathyroidism, sarcoidosis, tuberculosis of the kidney and AIDS-related infections, vitamin D toxicity as well as the use of certain drugs, such as acetazolamide, amphotericin B, triamterene and more rarely diuretics such as furosemide which has been described especially in premature infants (3, 4). Besides, any diuretic that acts proximal to the potassium secretory site will lead to increase urinary potassium excretion. The incidence of hypokalemia are dose dependent and are more frequent with thiazide diuretics than with loop diuretics, besides thiazides decrease calcium excretion while loop diuretics significantly increase calcium excretion (5).

Our patient presented chronic hypokalemia, metabolic alkalosis with elevated urinary Na, Cl, K and Ca, normotension despite hyperreninism and hyperaldostreonism(normal ratio), accompanied by nephrocalcinosis, and a history of postpartum anxious depressive syndrome after her first pregnancy. In our case, diuretic levels were detected in a patient in whom these drugs have not been prescribed. It is sometimes difficult to distinguish between tubulopathy (Bartter or Gitelman syndrome) and chronic surreptitious diuretic intake. In this clinical context, the determination of diuretics in urine or serial urinary electrolytes at different times of the same day (in chronic diuretic intake urine ions will not be persistently elevated, only after diuretics intake), can help us to distinguish these situations since in Bartter's syndrome urinary ion elimination has little or no fluctuations. In our case, medullary nephrocalcinosis could be related to diuretic abuse (2).

Nephrocalcinosis, therefore, may be attributed to a markedly fluctuant calciuretic effect of diuretics. Besides, nephrocalcinosis has been reported in patients with Bartter’s syndrome, which may suggest that both conditions have similar pathogenesis. Hypercalciuria is not a constant finding in Bartter’s syndrome, as in patients with furosemide abuse (2). Persistent alkalosis and alkaline urine secondary to chloride deficiency could result in nephrocalcinosis in both Bartter’s syndrome and patients. with furosemide abuse. Hypokalaemic metabolic alkalosis during furosemide abuse may also lead to the precipitation of calcium phosphate (6). Discrete hyperaldosternism may result from the response to volume depletion. 

The risk of nephrocalcinosis have been correlated with the daily dose of furosemide (2). Furosemide-associated nephrocalcinosis has not been fully recognized in adult patients in contrast with premature infants.

Surreptitious diuretic intake is a clinical condition to rule out in patients with chronic hypokalemia, metabolic alkalosis with elevated urinary sodium and chloride. The relation between surreptitious diuretic intake and nephrocalcinosis has not been fully elucidated in adults.
